# Langerhans Cell Histiocytosis Presenting as Uncontrolled Asthma

**DOI:** 10.1155/2013/637232

**Published:** 2013-08-27

**Authors:** Frederic A. Rawlins, James E. Hull, Julia A. Morgan, Michael J. Morris

**Affiliations:** ^1^Internal Medicine Residency, Department of Medicine (MCHE-MDX), San Antonio Military Medical Center, 3551 Roger Brooke Drive, Fort Sam Houston, TX 78234-6200, USA; ^2^Pulmonary/Critical Care Service, Department of Medicine (MCHE-MDX), San Antonio Military Medical Center, 3551 Roger Brooke Drive, Fort Sam Houston, TX 78234-6200, USA

## Abstract

Langerhans cell histiocytosis (LCH) is an uncommon disorder affecting primarily young adult smokers. It is characterized by abnormal proliferation of Langerhans cells, specialized monocyte-macrophage lineage antigen-presenting cells. LCH can affect the lungs in isolation or as part of a systemic disease. Most commonly, the disease presents in the third or fourth decade without gender predominance. Symptoms typically include dyspnea and cough. Commonly, physical examination is unremarkable but cor pulmonale may be observed in advanced disease. The chest radiograph is typically abnormal with nodular or interstitial infiltrates and cystic changes. High-resolution computed tomography of the chest with these findings in the middle and upper lobes of an adult smoker is virtually diagnostic of LCH. Pulmonary function assessment is variable. Asthma has rarely been reported in association with this disorder. There are only three reported cases of the diagnosis of concomitant asthma which have been made in association with the diagnosis of LCH. We present a case in which our patient presented with signs and symptoms of asthma to include confirmatory findings of airway hyperresponsiveness. The diagnosis of LCH was established after the patient failed to respond to conventional treatment for asthma, and further evaluation was completed.

## 1. Introduction

Langerhans cell histiocytosis (LCH) is an uncommon disorder affecting primarily young adult smokers [1–3]. It is characterized by abnormal proliferation of Langerhans cells, specialized monocyte-macrophage lineage antigen-presenting cells. Langerhans cell histiocytosis can affect the lungs in isolation or as part of a systemic disease affecting the bones, pituitary, liver, lymph nodes, or thyroid gland. When involving the lungs, the disorder is typically referred to as pulmonary LCH, pulmonary eosinophilic granuloma, or pulmonary histiocytosis X. As a systemic disorder, it can be referred to as Letterer-Siwe or Hand-Schüller-Christian disease. Most commonly, the disease presents in the third or fourth decade without gender predominance. Pulmonary LCH is typically characterized by dyspnea and cough and more than 90% of patients with pulmonary LCH are current smokers. Commonly, physical examination is unremarkable although evidence of cor pulmonale may be observed in advanced disease. The chest radiograph is typically abnormal with findings to include nodular or interstitial infiltrates and cystic changes [4–6]. High-resolution computed tomography (HRCT) of the chest with the appearance of diffuse irregularly shaped cysts and peribronchiolar nodular opacities in the middle and upper lobes of an adult smoker is virtually diagnostic of LCH [7]. Ground glass attenuation, adenopathy, and cystic changes involving the lower lobes may also be noted on HRCT. Pulmonary function assessment is variable and may reveal either a normal, restrictive, obstructive, or mixed pattern based on disease severity [4–6]. The most consistent finding on pulmonary function testing (PFT) is a decreased diffusion capacity for carbon monoxide (DLCO) [6, 8]. Asthma has rarely been reported in association with this disorder. There are only three reported cases of the diagnosis of concomitant asthma which have been made in association with the diagnosis of LCH [9–11]. We present a case in which our patient presented with signs and symptoms of asthma to include confirmatory PFT findings of AHR. The diagnosis of LCH was established after the patient failed to respond to conventional treatment for asthma and further evaluation was completed.

## 2. Case Report

**Figure 1:**
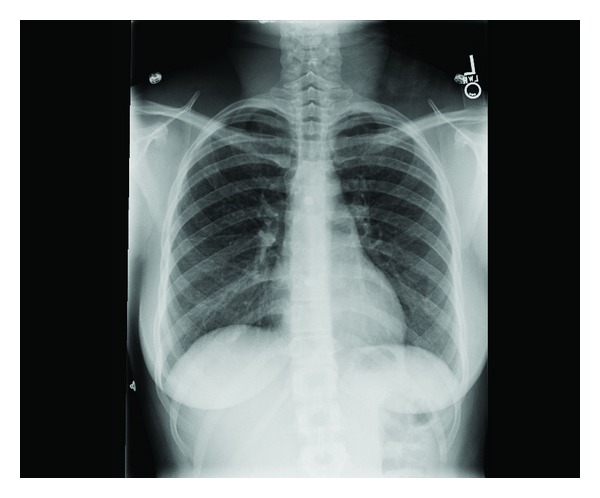
Chest X-ray.

**Figure 2:**
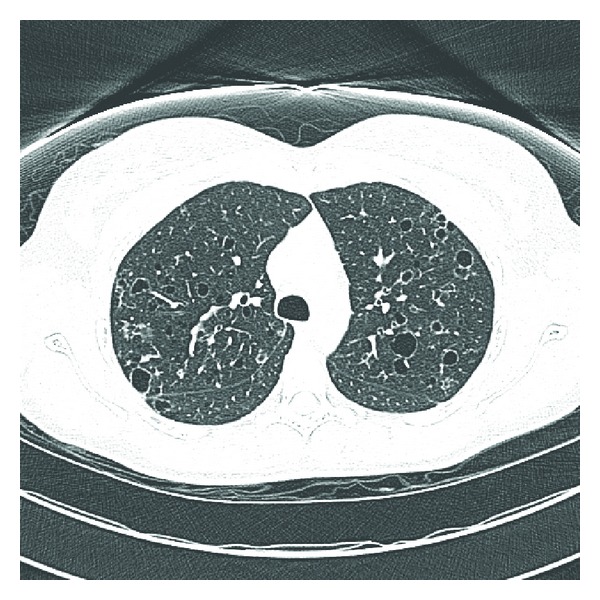
High-resolution chest CT.

**Figure 3:**
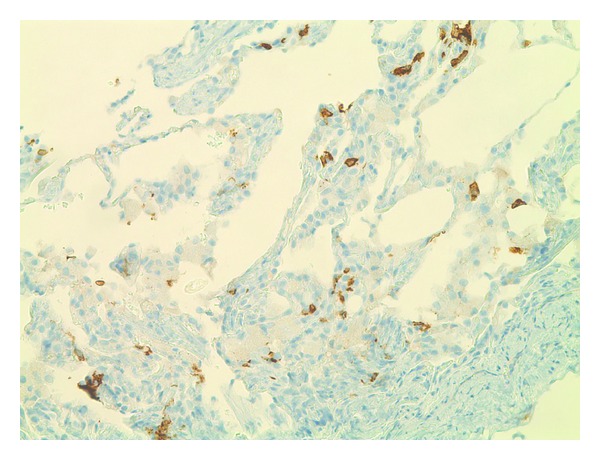
CD1a antigen staining.

**Figure 4:**
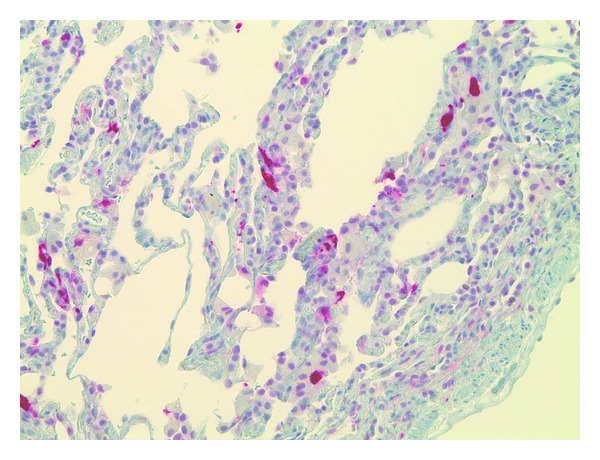
S100 protein staining.

A 26-year-old Dominican born active duty military female presented to a primary care clinic with complaints of dyspnea, wheezing, and chest tightness that were exacerbated by running. She had intermittently smoked over the past 10 years about 3–5 cigarettes per day with increased use up to a half-pack per day prior to presentation. She was previously healthy with a preliminary diagnosis of asthma one year prior to presentation requiring as needed albuterol for symptom control. Physical examination was notable for bilateral end-expiratory wheezing. Her initial spirometry was within normal limits to include a forced vital capacity (FVC) of 3.93 (91% predicted), a forced expiratory volume at one second (FEV_1_) of 2.89 (85% predicted), and FEV_1_/FVC ratio of 87%. There was a 10% increase in FEV_1_ to 3.18 (94% predicted) after bronchodilator. The patient was initially treated for asthma with daily combination medium-dose salmeterol/fluticasone inhaler and levalbuterol as needed. At her initial followup, she reported increasing symptoms and was referred to our pulmonary clinic for further evaluation. Chest radiograph (Figure 1) demonstrated no cardiopulmonary abnormalities and repeat spirometry was unchanged from baseline. Due to her symptoms and partial bronchodilator response, methacholine challenge testing was performed and was positive at 8 mg with a decrease in FEV_1_ from 106% at baseline to 79%. The patient had mildly elevated immunoglobulin E at 51.3 IU/mL. Her therapy was intensified with an increased dose of the inhaled fluticasone, and addition of a proton pump inhibitor and histamine-2 antagonist as well as daily montelukast. The patient noted improvement on this regimen but continued to have significant exertional dyspnea. Full PFT on inhaled medications demonstrated a normal FEV_1_ of 3.16 (94% predicted), FVC of 3.87 (99% predicted), and FEV_1_/FVC at 82% predicted. Total lung capacity was 4.50 (88% predicted) without an increase in residual volume and DLCO was notably decreased to 57% predicted (corrected to 69% for her measured hemoglobin). Due to continued dyspnea and the isolated reduction in DLCO, she underwent HRCT of the chest (Figure 2), which demonstrated multiple thin-walled air cysts with scattered three-millimeter nodules predominant throughout the upper lobes. Fiberoptic bronchoscopy with transbronchial biopsies revealed interstitial predominance of histiocytes with 80% of macrophages staining positively for CD1a and S100 and confirmed the diagnosis of pulmonary LCH (Figures 3 and 4). The patient was referred early in her treatment course for smoking cessation and was able to completely quit cigarette smoking within one year. In her most recent follow-up appointment, the patient had noted stable symptoms without significant change in PFTs or chest imaging while being maintained on her current pulmonary medications.

## 3. Discussion

A clinical presentation consistent with asthma or the presence of AHR is rarely associated with LCH. Large case series classically described the paucity of atopy or typical asthma symptoms such as expiratory wheezing in patients with LCH [4]. However, in a case series of 12 patients with confirmed LCH, 7 had evidence of AHR based on bronchoprovocation testing with carbachol [5]. The series did not report the baseline spirometry or bronchodilator response of these patients compared to other subjects. Our patient did have normal total lung capacity, no reduction in expiratory flow, and a decreased DLCO, which are the most common findings on PFTs [6]. However, our patient also manifested the presence of wheezing, a partial bronchodilator response based on FEV_1_.

Pulmonary function testing in LCH can vary between obstructive, restrictive, or mixed indices. Case series of LCH have determined that a decreased DLCO is the most consistent finding identified in 59–87% of reported cases [5, 6, 8, 12]. Obstructive patterns in LCH patients are reported in 20–50% of cases while restrictive patterns are 23–26% of cases [6, 8, 12, 13] but may be observed in 50% of LCH patients [5]. Restrictive patterns are more typically associated with advanced age and disease. The presence of cystic lung disease on HRCT correlates with abnormal PFT findings. This includes all patterns of decreased DLCO, obstruction, and restriction. Furthermore the burden of cystic changes can correlate with the severity of these findings when statistically analyzed [12].

Three cases have been identified in the medical literature in which patients were initially considered to have asthma and later diagnosed with LCH. The first case was a 21-year-old female diagnosed with cough-variant asthma who was found to have cystic changes on chest radiograph [9]. No information was presented on the patient's PFTs or evidence of AHR to confirm the clinical diagnosis of asthma. The second case described a 2-year-old female who was initially treated with bronchodilators and steroids for presumed asthma. The patient eventually presented with acute respiratory failure, and a chest radiograph identified reticulonodular infiltrates and cystic changes consistent with LCH. There was no documentation of AHR as this was a pediatric patient [10]. The third case was a nonsmoking 18-year-old male with 10-year history of respiratory symptoms treated with inhaled asthma medications for four years. He was diagnosed with LCH by computed tomography and surgical lung biopsy after presenting with a spontaneous pneumothorax [11]. Pulmonary function testing did reveal the presence of baseline obstruction, a 12% response to bronchodilator, and hyperinflation. AHR was confirmed with methacholine challenge testing at 8 mg/mL after which the patient was treated with inhaled corticosteroid and reportedly well at 1-month followup.

These cases illustrate the diagnostic complexity of this LCH as patients often have a delay in diagnosis for years. Our patient had a normal baseline chest radiograph, wheezing on examination, and a 10% bronchodilator presence with normal spirometric indices. Our patient likewise had evidence of AHR during methacholine challenge testing. The lack of clinical response to asthma medications prompted further testing which identified a low DLCO (not typical for asthma) and a characteristic HRCT for LCH. Obstructive indices can be identified in LCH and AHR may also be found in these patients. This case affirms that a secondary evaluation should be undertaken when patients fail to respond to appropriate therapy for asthma. While asthma is common in the age group affected by LCH, there is a relative paucity of reported cases of asthma associated with LCH. This raises the question of whether LCH is protective against the development of asthma. Alternatively, the diagnosis of coexisting asthma may be underrecognized. We suggest this relationship to be a subject of future study.

## Abbreviations

LCH: Langerhans cell histiocytosis
HRCT: High-resolution computed tomography
PFT: Pulmonary function testing
DLCO: Diffusion capacity for carbon monoxide
AHR: Airway hyperresponsiveness
FVC: Forced vital capacity
FEV_1_: Forced expiratory volume at one second.
